# ﻿*Achnanthidiumgladius* sp. nov. (Bacillariophyceae) – a new monoraphid diatom species from Indonesia

**DOI:** 10.3897/phytokeys.187.73913

**Published:** 2021-12-17

**Authors:** Natalia D. Tseplik, Yevhen I. Maltsev, Anton M. Glushchenko, Irina V. Kuznetsova, Sergei I. Genkal, Evgeniy S. Gusev, Maxim S. Kulikovskiy

**Affiliations:** 1 K.A. Timiryazev Institute of Plant Physiology RAS, IPP RAS, 35 Botanicheskaya St., Moscow, 127276, Russia; 2 I.D. Papanin Institute for Biology of Inland Waters, Russian Academy of Sciences, Yaroslavl, Nekouz, Borok, 152742, Russia

**Keywords:** *Achnanthidium*, diatoms, Indonesia, new species, molecular investigation

## Abstract

A new monoraphid diatom species *Achnanthidiumgladius***sp. nov.** is described from Indonesia. The description is based on molecular data (18SV4), morphological analysis and comparison with similar species. According to molecular data, *Achnanthidiumgladius* sp. nov. is closely related to *Achnanthidiumminutissimum*. Morphologically, the new species differs from similar species by the absence of a fascia on raphe valve, cell size, and striae density and pattern. The new species is only known from the type locality in Indonesia. Comparison with close related species is given.

## ﻿Introduction

The genus *Achnanthidium* Kützing is one of the largest genera of monoraphid diatoms. It contains more than two hundred species which are widely distributed worldwide in various types of water bodies. *Achnanthidium* was first suggested by F. Kützing (1844), who included in it all monoraphid species that do not form colonies. Later, P.T. Cleve (1895) changed the status of this taxon and made it a subgenus of *Achnanthes* s.l. Most of monoraphid diatoms were included in *Achnanthes* s.l., and this system was used by many authors for a long time.

Round et al. (1990) reinstated *Achnanthidium* as a separate genus, with the explanation that *Achnanthes* and *Achnanthidium* differ significantly in the structure of areolae, girdle, raphe, and plastids. Round and Bukhtiyarova (1996) suggested an improved diagnosis for the genus which included the following features: radiate uniseriate striae, small linear-lanceolate to elliptic-lanceolate valves, external distal raphe ends straight or curved to one side, and sternum widened at the centre. The species that belonged to *Achnanthidium* but did not correspond with this diagnosis were moved to other genera.

Since the beginning of the 21^st^ century many descriptions of new *Achnanthidium* species have been published (Monnier et al. 2004; Cantonati and Lange-Bertalot 2006; Ivanov and Ector 2006; Potapova 2006; Wojtal et al. 2010; Liu et al. 2021). Furthermore, the type materials of earlier described species are studied using light microscopy (LM) and scanning electron microscopy (SEM) (Hlúbiková et al. 2011; Van de Vijver et al. 2011). This data is used for clearer definition of species boundaries and separation of species complexes. Also, there have been attempts to separate species complexes using morphometric methods (Potapova and Hamilton 2007) and a complex of morphological, ecological and geographic studies (Jüttner et al. 2011).

Many authors note that the taxonomy of *Achnanthidium* is quite complicated for a number of reasons. First of all, most species of this genus are quite small, which makes light microscopy studies more difficult. Most of the features used for species identification are ultrastructural, so that SEM is required for precise identification. Also, the species boundaries may be unclear, since there are no criteria for species separation and values of quantitative features often overlap in different species, which complicates identification even further. Moreover, many species are quite similar in terms of morphology and require the usage of molecular methods. There are few molecular studies of *Achnanthidium* species. In a recent article (Pinseel et al. 2017) 12 different lines have been identified in the *Achnanthidiumminutissimum* (Kützing) Czarnecki species complex on the basis of molecular studies. The representatives of one of these lines have been described as a separate species *Achnanthidiumdigitatum* Pinseel, Vanormelingen, Hamilton & Van de Vijver (Pinseel et al. 2017).

In Indonesia, *Achnanthidium* has not been studied extensively. Most existing works concern the ecology and general biodiversity of diatoms in different water bodies (Hustedt 1937; Bramburger et al. 2004; Sabo et al. 2008; Soeprobowati et al. 2016) but not the taxonomy of the genus. Indonesia is a less studied region with a high level of endemicity (Bramburger et al. 2004; Kapustin et al. 2017, 2019, 2020; Kapustin and Kulikovskiy 2018; Kociolek et al. 2018; Kulikovskiy et al. 2020), which indicates a high probability of finding new diatom species there.

## ﻿Materials and methods

### ﻿Sample collection

The sample used in this study was collected from Indonesia by Ivanov I.I. on 23.09.2011. It was designated as no. I241 and was collected from the Lake Matano (02°31.985'S, 121°26.279'E), epipsammon, pH 8.36, conductivity 187 μS cm^-1^.

### ﻿Culturing

Monoclonal strains were established by micropipetting single cells under an inverted microscope Zeiss Axio Vert. A1. The culture was maintained in the liquid medium WC (Guillard and Lorenzen 1972) in a lightbox with the photoperiod day:night 12:12 hours and temperature = 22–25 °C. The culture was grown for a month. The strain was designated Ind391.

### ﻿Preparation of slides and microscopic observation

The monoclonal culture was boiled in 30% hydrogen peroxide at the temperature 150–160 °C for 8 hours to dissolve organic matter. After decanting and refilling up to 100 ml with deionized water, the suspension was spread onto coverslips and left to dry at room temperature. Permanent diatom preparations were mounted in Naphrax (refraction index=1.73). LM observations were performed with a Zeiss Axio Scope.A1 microscope equipped with an oil immersion objective (×100, n.a. 1.4, differential interference contrast) and Axio Cam ERc 5s camera. Valve ultrastructure was examined using a JEOL JSM-6510LV scanning electron microscope.

### ﻿Molecular investigation

Total DNA of the strain Ind391 was extracted using HelixTM (Bio-Rad Laboratories, USA) according to the manufacturer’s protocol. A fragment of 18S rDNA (435 bp, including V4 domain) was amplified using primers D512 and D978 (Zimmermann et al. 2011). Amplification of the 18S rDNA fragment was carried out using the premade mix ScreenMix (Evrogen, Russia) for the polymerase chain reaction (PCR). The conditions of amplification for 18S rDNA fragment were: an initial denaturation of 5 min at 95 °C, followed by 35 cycles: denaturation at 94 °C (30 s), annealing at 52 °C (40 s), elongation at 72 °C (50 s); and a final extension of 5 min at 72 °C.

The resulting amplicons were visualized by horizontal agarose gel electrophoresis (1%), colored by SYBR Safe (Life Technologies, United States). Purification of DNA fragments was performed with the mix of FastAP, 10× FastAP Buffer, Exonuclease I (Thermo Fisher Scientific, USA) and water. 18S rDNA fragment was decoded from two sides using forward and reverse PCR primers and the Big Dye system (Applied Biosystems, USA), followed by electrophoresis using a Genetic Analyzer 3500 sequencer (Applied Biosystems).

**Figure 1.**
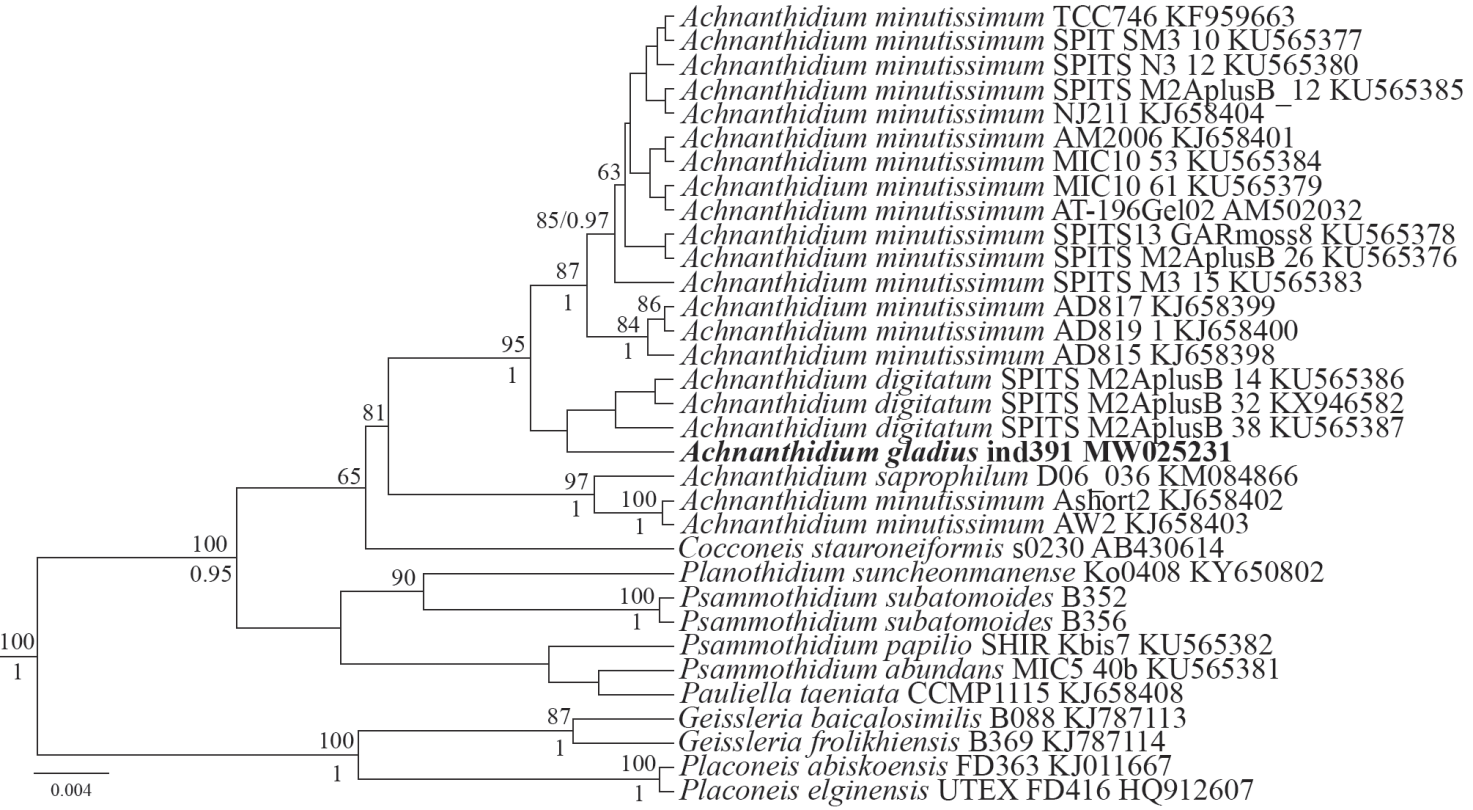
Phylogenetic position of *Achnanthidiumgladius* Ind391 (indicated in bold) based on Bayesian inference for the partial 18S rDNA gene. Total length of the alignment is 441 characters. Bootstrap supports from ML (constructed by RaxML) are presented above the horizontal lines (slash). Posterior probabilities from BI (constructed by Beast) are presented below the horizontal lines (slash). Only BS and PP above 50 and 0.9 are shown. All sequences have strain numbers (if available) and GenBank numbers.

Editing and assembling of the consensus sequences were carried out by comparing the direct and reverse chromatograms using the Ridom TraceEdit program (ver. 1.1.0) and Mega7 (Kumar et al. 2016). Newly determined sequence and DNA fragments from 32 other diatoms, which were downloaded from GenBank (taxa and Accession Numbers are given in Fig. 1), were included in the alignments. Diatom species from genera *Geissleria* Lange-Bertalot & Metzeltin and *Placoneis* Mereschkowsky were chosen as the outgroup. The nucleotide sequences of the 18S rDNA gene were aligned separately using the Mafft v7 software and the E-INS-i model (Katoh and Toh 2010). The resulting alignment had the length of 441 characters.

The dataset was analyzed using the Bayesian interference (BI) method implemented in Beast ver.1.10.1 (Drummond and Rambaut 2007) to construct phylogeny. For each of the alignment partitions, the most appropriate substitution model was estimated using the Bayesian information criterion (BIC) as implemented in jModelTest 2.1.10 (Darriba et al. 2012). This BIC-based model selection procedure selected HKY+I+G model, shape parameter α = 0.5800 and a proportion of invariable sites (pinvar) = 0.7460. A Yule process tree prior was used as a speciation model. The analysis ran for 15 million generations with chain sampling every 100 generations. The parameters – estimated convergence, effective sample size (ESS) and burn-in period were checked using the software Tracer ver. 1.7.1. (Drummond and Rambaut 2007). The initial 25% of the trees were removed, the rest remained to reconstruct a final phylogeny. The phylogenetic tree and posterior probabilities of its branching were obtained on the basis of the remaining trees, having stable estimates of the parameter models of nucleotide substitutions and likelihood. Maximum Likelihood (ML) analysis was performed using the program RA×ML (Stamatakis et al. 2008). The nonparametric bootstrap analysis with 1000 replicates was used. The statistical support values were visualized in FigTree (ver. 1.4.2) and Adobe Photoshop CC (19.0).

## ﻿Results

Figure 1

The molecular analysis has established that the strain *Achnanthidiumgladius* sp. nov. belongs to a group of close species that includes *A.minutissimum* and *A.digitatum* (ML 87/BI 100). A branch that includes three strains of two species (*Achnanthidiumminutissimum* and *A.saprophilum*) is also close (ML 87/BI 100). Overall, the new species belongs to a large clade of monoraphid and cymbelloid taxa from such genera as *Cocconeis*, *Pauliella*, *Psammothidium*, *Planothidium*, *Geissleria*, *Placoneis*, and others.

The morphological description of the new species is given below.

### ﻿ Achnanthidium gladius

Taxon classificationPlantaeCocconeidalesAchnanthidiaceae

Tseplik, Kulikovskiy, Glushchenko & Genkal sp. nov.

9FCA09E0-E304-555E-8739-D0D09BD664CC

Figures 2 , 3

#### Holotype.

**Figure 2.**
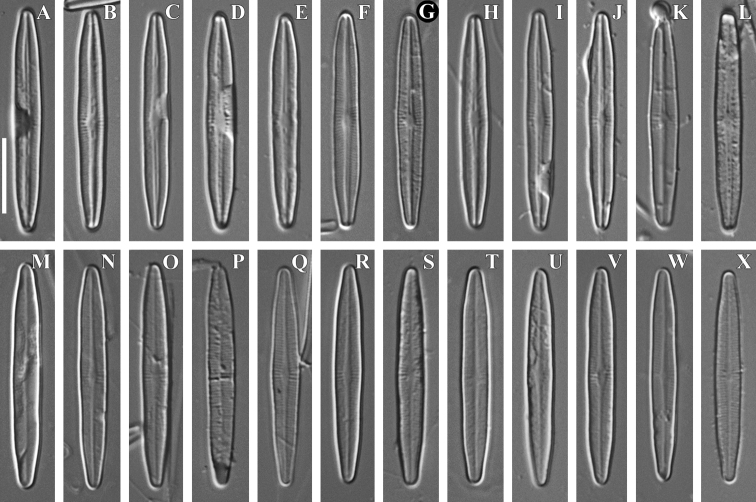
**A–X***Achnanthidiumgladius* Tseplik, Kulikovskiy, Glushchenko & Genkal, sp. nov. LM, DIC, size diminution series. Slide no 04123. **A–L** raphe valves **M–X** rapheless valves. Holotype (**G**). Scale bar: 10 μm.

Slide no 04123 in collection of MHA, Main Botanical Garden Russian Academy of Science, Moscow, Russia, represented here by Fig. 2G.

#### Reference strain.

Strain Ind391, isolated in sample no. I241.

**Figure 3.**
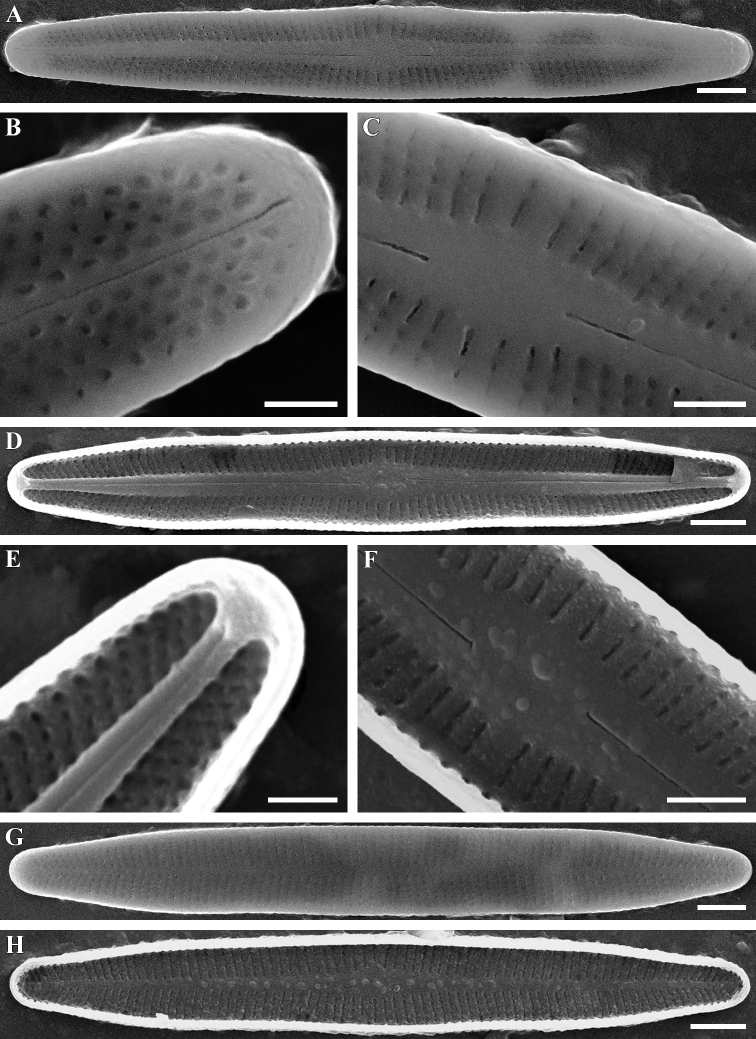
**A–H***Achnanthidiumgladius* Tseplik, Kulikovskiy, Glushchenko & Genkal, sp. nov. SEM. Sample no 04123. **A–F** raphe valves **G, H** rapheless valves **A–C, G, H** external views **D–F, H** internal views. Scale bars: 2 μm (**A, D, G, H**), 1 μm (**C, F**), 0.5 μm (**B, E**).

#### Type locality.

Indonesia, Lake Matano, 02°31.985'S, 121°26.279'E.

#### Description.

***LM*** (Fig. 2). Valves relatively big, linear to linear-lanceolate, with gradually narrowing, rounded apices. Length 27.7–30.4 µm, breadth 3.3–4.0 µm. Raphe straight, filiform. Striae on the raphe valve radiate, uniseriate, 27–28 in 10 µm, consist of round and narrow elongated areolae. On the rapheless valve the central and axial areas are absent. Striae are almost parallel, 26–27 in 10 µm, becoming noticeably more radiate near the valve apices. Areolae not resolved in LM.

***SEM*** (Fig. 3). External proximal raphe ends simple, distal raphe ends straight. Internal proximal raphe ends slightly bent in opposite directions, distal raphe ends terminate is weakly developed helictoglossae. Raphe positioned in a narrow axial area that slightly widens near the centre of the valve. A distinct central area is absent, but 2 or 3 striae in the center of the valve are spaced wider than the majority of the striae. Areolae are mostly elongated.

#### Etymology.

The specific epithet “*gladius*” refers to the similarity in valve morphology contour with a sword.

#### Distribution.

As yet known only from the type locality.

#### Sequence data.

Partial 18S rDNA gene sequence comprising V4 domain sequence (GenBank accession number MW025231).

## ﻿Discussion

The new species *Achnanthidiumgladius* sp. nov. belongs to the genus *Achnanthidium* and possesses the characteristic features of the genus, including a linear-lanceolate valve shape, radiate uniseriate striae and straight external distal raphe ends. *Achnanthidium* species are divided into two morphological groups: the *A.minutissimum* species complex has straight external distal raphe ends, and the *Achnanthidiumpyrenaicum* (Hustedt) Kobayasi species complex has external distal raphe ends that are distinctly curved to one side. Since *A.gladius* sp. nov. has straight external distal raphe ends, it belongs to the *A.minutissimum* species complex. The new species has quite large valves, which is characteristic only for several of the known *Achnanthidium* species (Table 1).

**Table 1.** Comparison of new species with similar taxa of *Achnanthidium*.

	Outline	Valve apices	Axial area	Central area	Valve length (μm)	Valve width (μm)	Striae in raphe valve (in 10 μm)	Striae in rapheless valve (in 10 μm)	Distribution	Reference
*A gladius* sp. nov.	relatively big, linear to linear-lanceolate	gradually narrowing, rounded	absent	absent, but 2 or 3 striae in the center of the valve are spaced wider than the majority of the striae	27.7–30.4	3.3–4.0	27–28	26–27	Indonesia: Lake Matano	This study
*A.initium*	linear-lanceolate to lanceolate	rounded to weakly rostrate rounded	slightly expanded into asymmetrical transverse fascia at central area at the raphe valve; narrow linear at the rapheless valve	asymmetrical transverse fascia at the raphe valve; slightly expanded at the rapheless valve	11.5–25.5	9.0–13.0	29–34	32–35	India: Masilla Waterfalls	Karthick et al. 2017
*A.sublanceolatum*	linear-lanceolate	rounded or weakly protracted	narrow, linear-lanceolate	rounded at the raphe valve; slightly expanded at the rapheless valve	18–35	4.0–4.5	20–23 at the middle portion, 36–42 near the apices	21–24 in the center, and 30–36 near the apices	China: Taiping Lake	Yu et al. 2019
*A.standeri*	not or very slightly dorsiventral, subelliptical-lanceolate, dorsal and ventral margins moderately arched	protracted, apiculate to rostrate	narrow, linear, narrowing slightly towards to the ends, almost median line of the valve	irregular, asymmetric space of different extent	8–37	2.8–4.4	28–30	24–26	South Africa	Taylor et al. 2011

There are some known *Achnanthidium* species that are morphologically similar to *A.gladius* sp. nov. The most similar is *Achnanthidiuminitium* Karthick, Taylor & Hamilton (Karthick et al. 2017); however, it can be differentiated from the new species by several features. The most obvious one is the fascia on the raphe valve of *A.initium*, while *A.gladius* sp. nov. lacks a central area; also *A.initium* has external distal raphe ends that are curved to opposite sides. Furthermore, these species differ in valve length (27.7–30.4 μm in *A.gladius* sp. nov., 11.5–25.5 μm in *A.initium*) and the striae density on both raphe and rapheless valves (raphe valves: 27–28 in 10 μm in *A.gladius* sp. nov., 29–34 in 10 μm in *A.initium*; rapheless valves: 26–27 in 10 μm in *A.gladius* sp. nov., 32–35 in 10 μm in *A.initium*).

Two more similar species are *Achnanthidiumsublanceolatum* Yu, You & Kociolek (Yu et al. 2019) and *Achnanthidiumstanderi* Taylor, Morales & Ector (Taylor et al. 2011). *A.sublanceolatum* is similar to the new species in valve shape and size, but has lower striae density (20–23 in 10 μm on the raphe valve, 21–24 in 10 μm on the rapheless valve) and curved external distal raphe ends. *A.standeri*, as well as the new species, belongs to the *A.minutissimum* species complex. It can be differentiated by the fascia on the raphe valve and radiate striae on the rapheless valve.

On the phylogenetic tree the strain *A.gladius* sp. nov. belongs to the clade that includes a lot of *A.minutissimum* strains. The strain used in the present study forms a cluster with three *A.minutissimum* strains, but is separated from them with high statistical support, which indicates that our strain is a separate species. Although *A.minutissimum* and *A.gladius* sp. nov. are closely related, morphologically they are significantly different, which confirms the identification of *A.gladius* sp. nov. as a separate species. The group that includes the new species is sister to a cluster of *A.digitatum* strains, which was recently separated from *A.minutissimum*.

Extensive molecular investigations are required for better understanding of taxonomy in the genus *Achnanthidium* and the *A.minutissimum* species complex. Often it is impossible to separate species of the genus without molecular methods. However, molecular data is available for a very small number of *Achnanthidium* species: GenBank has sequences for 13 identified species and several sequences that are labeled as *Achnanthidium* sp. Including DNA sequences in new species descriptions makes the subsequent identification of these species much easier, and also contributes to the establishment of a database of various *Achnanthidium* strains.

## Supplementary Material

XML Treatment for Achnanthidium gladius
